# Genome-wide association reveals a locus in neuregulin 3 associated with gabapentin efficacy in women with chronic pelvic pain

**DOI:** 10.1016/j.isci.2024.110370

**Published:** 2024-07-15

**Authors:** Scott C. Mackenzie, Nilufer Rahmioglu, Liana Romaniuk, Frances Collins, Lydia Coxon, Heather C. Whalley, Katy Vincent, Krina T. Zondervan, Andrew W. Horne, Lucy H.R. Whitaker

**Affiliations:** 1 Centre for Reproductive Health, Institute for Regeneration and Repair, University of Edinburgh, Edinburgh EH16 4UU, UK; 2 Wellcome Centre for Human Genetics, University of Oxford, Old Road Campus, Roosevelt Drive, Oxford OX3 7BN, UK; 3 Oxford Endometriosis CaRe Centre, Nuffield Department of Women’s & Reproductive Health, University of Oxford, John Radcliffe Hospital, Oxford OX3 9DU, UK; 4 Division of Psychiatry, University of Edinburgh, Edinburgh EH10 5HF, UK; 5 Generation Scotland, Institute for Genetics and Cancer, University of Edinburgh, Edinburgh EH4 2XU, UK

**Keywords:** Association analysis, Neurogenetics, Pain management in health technology, Quantitative genetics

## Abstract

Chronic pelvic pain (CPP) in women with no obvious pelvic pathology has few evidence-based treatment options. Our recent multicenter randomized controlled trial (GaPP2) in women with CPP and no obvious pelvic pathology showed that gabapentin did not relieve pain overall and was associated with more side effects than placebo. We conducted an exploratory genome-wide association study using eligible GaPP2 participants aiming to identify genetic variants associated with gabapentin response. One genome-wide significant association with gabapentin analgesic response was identified, rs4442490, an intron variant located in Neuregulin 3 (*NRG3*) (*p* = 2·11×10^−8^; OR = 18·82 (95% CI 4·86–72·83). Analysis of a large sample of UK Biobank participants demonstrated phenome-wide significant brain imaging features of rs4442490, particularly implicating the orbitofrontal cortex. *NRG3* is expressed predominantly in central nervous system tissues and plays a critical role in nervous system development, maintenance, and repair, suggesting a neurobiologically plausible role in gabapentin efficacy and potential for personalized analgesic treatment.

## Highlights

• Gabapentin response GWAS was performed in a cohort of women with chronic pelvic pain
• A locus in Neuregulin 3 (*NRG3*) was associated with gabapentin analgesic efficacy
• *NRG3* is expressed in nervous system tissues and involved in regulation and repair
• There may be potential for a personalised medicine approach to gabapentin treatment

## Graphical abstract

**Figure:**
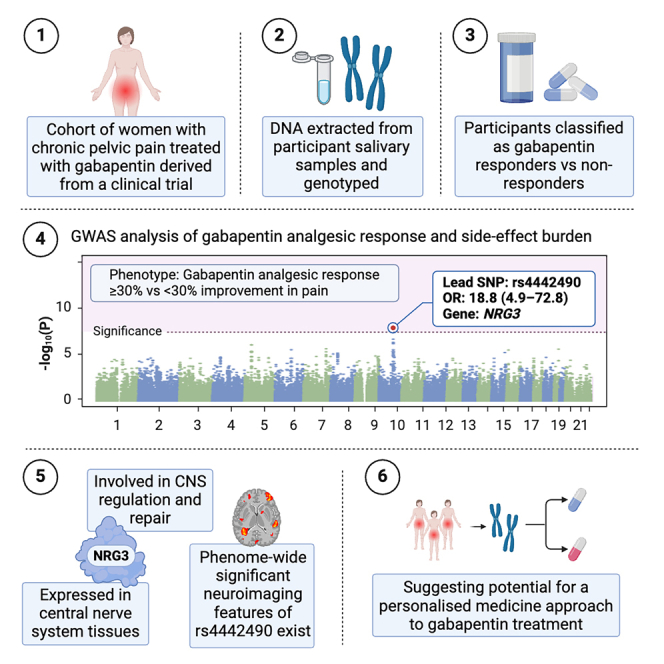

## Introduction

Chronic pelvic pain (CPP) in women and those assigned female at birth is a debilitating condition with an estimated prevalence of 26% worldwide.^1^ Like other chronic pain conditions, CPP is associated with a marked reduction in the quality of life and substantial economic and societal cost.^2^ Many women undergo laparoscopic investigation to identify an underlying cause for their symptoms such as endometriosis. However, at diagnostic laparoscopy, no underlying pathology is found in up to 55%.^3^ For those in whom no pathology is identified, learning of the idiopathic nature of their chronic pain and the lack of effective treatments can be difficult.

Although biological mechanisms driving “laparoscopy negative” CPP are poorly understood, evidence suggests that like in other chronic pain conditions, both central and peripheral nervous system (CNS and PNS respectively) sensitization can occur, leading to the development of a nociplastic or neuropathic-like pain component.^4^^,^^5^^,^^6^^,^^7^ Consequently, many women with CPP are prescribed gabapentin, a neuromodulator, that has proven efficacy in other chronic pain conditions.^8^ However, gabapentin gained popularity for the treatment of CPP despite an absence of high-quality evidence supporting its use in CPP specifically. These circumstances warranted further investigation of the efficacy of gabapentin for the treatment of CPP through a large clinical trial. The trial, *Gabapentin for chronic pelvic pain in women* (GaPP2), was a UK-wide, multicenter, randomized, controlled trial of gabapentin versus placebo for the treatment of CPP. The trial concluded that gabapentin was not superior to placebo in improving worst or average pain scores and was associated with more side effects.^9^ However, despite an overall lack of efficacy, the data suggest subgroups of women derive benefit. A total of 40% (*n* = 49/123) of women treated in the trial with gabapentin experienced a ≥30% improvement in worst and/or average pain, representing a “*moderate improvement.*”^10^ Considering gabapentin’s unfavorable side effect profile and the risk of dependence, predicting those who are likely to benefit, and stratifying treatment approach accordingly would be highly useful.

Gabapentin has substantial interindividual variability in both therapeutic response and vulnerability to adverse drug reactions. The variation in gabapentin efficacy is thought to be partially driven by pharmacokinetic factors such as dose-dependent oral bioavailability, drug transporter(s) activity, and influence of age or renal function.^11^ For other mainly opioid analgesics, common genetic variants are associated with drug efficacy, toxicity, and risk of dependence.^12^^,^^13^ Some groups have investigated gabapentin pharmacogenomics through hypothesis-driven (i.e., candidate gene or single nucleotide polymorphism (SNP)) approaches, identifying SNPs associated with anticonvulsant and analgesic treatment response in epilepsy and neuropathic pain respectively.^14^^,^^15^ The last decade has shown unbiased approaches such as pharmacogenomic genome-wide association studies have been successful, even with small sample sizes, in identifying genetic contributions to interindividual differences in drug response.^16^

In this study, using an exploratory genome-wide approach in a small and well-defined cohort of women with CPP and no obvious pelvic pathology, we identify a locus associated with gabapentin analgesic efficacy, that is both plausibly mechanistically involved and has the capacity to predict subsequent gabapentin response.

## Results

### Study participants

A total of 93 women who were randomized to receive gabapentin in the GaPP2 trial provided a saliva sample for inclusion in this study. Participants who were lost to follow-up and had missing endpoint pain scores or side effect data were excluded from further analysis, resulting in the exclusion of 16 and 17 individuals for pain response and side effect burden phenotypes, respectively. In addition, six and nine individuals were excluded after sample quality control, in the pain response and side effect groups respectively. This left 71 samples for pain response analysis (29 pain responders and 42 pain non-responders) and 67 samples for side effect burden analysis (46 with ≥3 side effects and 21 with <3 side effects). A flowchart of study participants is available as Figure S2.

Baseline characteristics of participants including baseline average and worst pain were similar among pain response and side effect burden groups (Tables 1 and 2). Within the pain responder group, pain scores reduced during the study period by an average of 47% and 34% for average and worst pain respectively. In the pain non-responder groups, pain scores increased during the study period by an average of 8% and 1% for average and worst pain respectively.

**Table 1:** Characteristics of participants included in final GWAS per gabapentin pain response phenotype

	Pain responder (*n* = 29)	Pain non-responder (*n* = 42)	p
Age (years)	31.8 (8.1)	29.6 (7.2)	0.26
BMI (kg/m^2^)	28.9 (7.7), 28	26.2 (4.9), 41	0.11
Side effects (n)	4 (2.3), 25	3.6 (2.3), 40	0.45
Average pain (NRS)			
Baseline	5.2 (1.7)	5·5 (1·7)	0.38
End of study	2.6 (1.4)	5·7 (1·6)	<0.001
Worst pain (NRS)			
Baseline	9 [1]	9 [1]	0.56
End of study	6 [3]	9 [1]	<0.001

Mean (SD); median [IQR], n where n is different from the total number of participants. NRS: numerical rating scale. BMI: body mass index.

**Table 2:** Characteristics of participants included in final GWAS per gabapentin side effect burden phenotype

	≥3 side effects (*n* = 46)	<3 side effects (*n* = 21)	P
Age (years)	31.3 (7·3)	28.4 (7.7)	0.16
BMI (kg/m^2^)	27.5 (6·6), 45	27.6 (5.7)	0.93
Side effects (n)	4.9 (1·6)	1.1 (0.9)	<0.001
Average pain (NRS)			
Baseline	5.5 (1·6)	5.3 (1.6)	0.57
End of study	4.6 (1.9), 44	4.4 (2.0)	0.70
Worst pain (NRS)			
Baseline	9 [1]	9 [2]	0.35
End of study	8 [4], 44	8 [4]	0.81

Mean (SD); median [IQR], n where n is different from the total number of participants. NRS: numerical rating scale. BMI: body mass index.

### Genome-wide association

**Figure 1:**
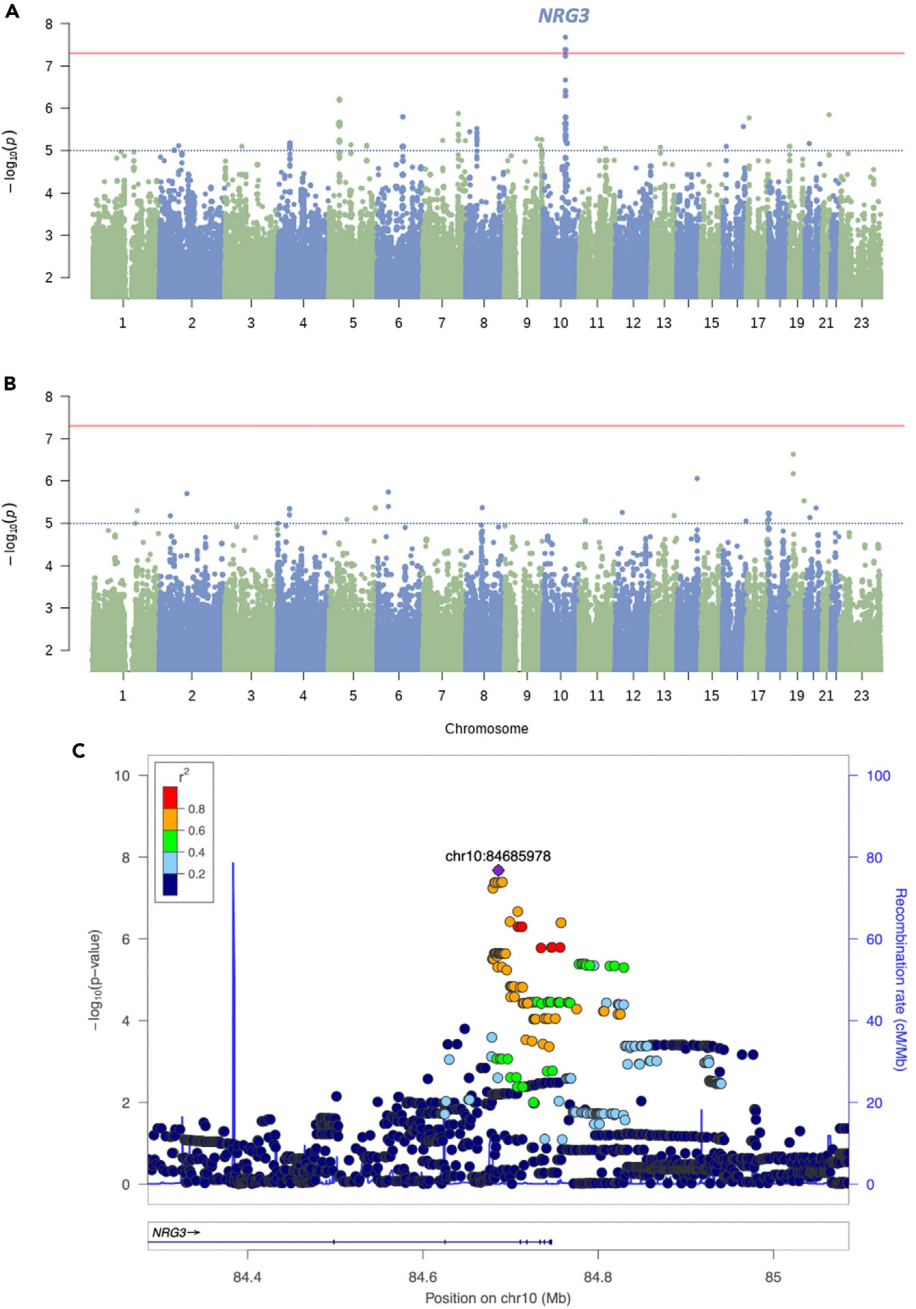
Manhattan plots and regional association plot of GWAS results (A) Manhattan plot of gabapentin pain response. (B) Manhattan plot of side effect burden. (C) Regional association plot gabapentin pain response lead SNP (rs4442490 (chr10:84685978)), generated using LocusZoom (http://locuszoom.org/). The genome-wide significance threshold (*p* < 5 × 10^−8^) is indicated by the red horizontal line and the nominal significance threshold (*p* < 1 × 10^−5^) is indicated by the blue dotted line.

After variant quality control and imputation, we analyzed a total of 5,522,729 SNPs for the association. We observed minimal evidence of population stratification for gabapentin pain response (λ = 1·046) or gabapentin side effect burden (λ = 1.033); QQ plots are available in Figure S3. Our analysis identified six SNPs in one locus that reached genome-wide significance for gabapentin pain response (see Figure 1). The lead SNP was rs4442490, an intronic variant located in Neuregulin 3 (*NRG3*) at 10q23·1 (*p* = 2.11 × 10^−8^; OR = 18.82 (95% CI 4·86–72·83)). Five neighboring SNPs in linkage disequilibrium (LD) with rs4442490 (r^2^ > 0.6) were observed at genome-wide significance within the same locus (see Table 3). Genotype distribution data is available in Table S1. No eQTL data was available in GTEx v8 for the six SNPs that reached genome-wide significance. No SNPs reached the genome-wide significance threshold for side effect burden.

**Table 3:** SNPs of genome-wide significance for gabapentin pain response

rsID	Chromosome	EA	NEA	EAF	EAF in gabapentin pain responders	EAF in gabapentin pain non-responders	p	OR (95% CI)
rs4442490	10	T	G	0.583	0.812	0.424	2.11 × 10^−8^	18.82 (4.86–72.83)
rs4933859	10	T	C	0.660	0.899	0.495	4.11 × 10^−8^	13.84 (4.34–44.12)
rs10885506	10	T	G	0.659	0.899	0.494	4.21 × 10^−8^	13.75 (4.32–43.71)
rs1923561	10	A	G	0.660	0.899	0.494	4.21 × 10^−8^	13.76 (4.33–43.77)
rs2026495	10	A	C	0.660	0.899	0.494	4.22 × 10^−8^	13.76 (4.33–43.75)
rs1923565	10	C	G	0.660	0.899	0.494	4.23 × 10^−8^	13.75 (4.32–43.73)

EA: effect allele; NEA: non-effect allele; EAF: effect allele frequency; OR: odds ratio; CI: confidence interval.

All six SNPs comprising the genome-wide significant locus in NRG3 were imputed with high imputation quality scores (>0·97). Of these, three SNPs (rs4442490, rs4933859, and rs2026495) were multiallelic, and the remaining three were biallelic. We also located 28 genotyped SNPs within 100kb upstream and downstream of the lead SNP (rs4442490), and three of these genotyped SNPs were in high LD (r^2^ > 0.6) with rs4442490. Intensity plots were generated for these three SNPs, which showed accurate genotype calling in discrete groups.

A genotypic test using rs4442490 predicted gabapentin pain response with a receiver operating characteristic (ROC) curve area under curve of 0·82 (95% CI 0·74–0·90; Figure S4).^17^

### Candidate gene analysis

**Figure 2:**
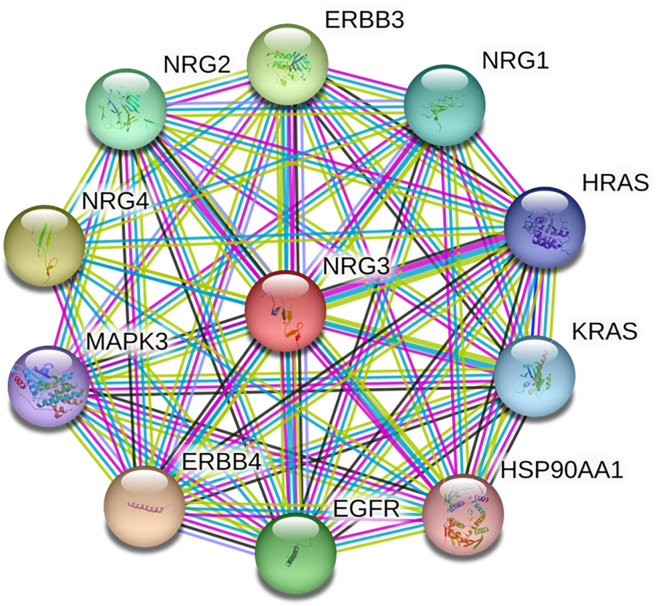
STRING (v11·5) network analysis for *NRG3* Candidate genes identified in network analysis with *NRG3* were tested for association with gabapentin analgesic efficacy. Network nodes represent proteins. Edges represent protein-protein associations. Color-coding of represents evidence category (green line: text mining evidence; purple line: experimental evidence; black line: co-expression evidence; light blue line: database evidence; blue line: cooccurrence evidence; lilac line: protein homology).

Candidate genes identified through literature review due to involvement with gabapentin pharmacology included *CACNA1C*,^18^ *KCNQ2*,^19^ *KCNQ3*,^19^ *KCNQ5*,^20^ *OCTN1*,^21^ and *OCT2*.^11^ No candidate gene SNP associations with gabapentin pain response or side effect burden were identified. Additionally, for gabapentin pain response, candidate genes identified through STRING network analysis with *NRG3* (*NRG1, NRG2, NRG4, ERBB3, ERBB4, KRAS, HSP90AA1, EGFR, MAPK3 and HRAS;* Figure 2) were tested for association; however, no significant SNP associations were identified.

### Functional mapping

**Figure 3:**
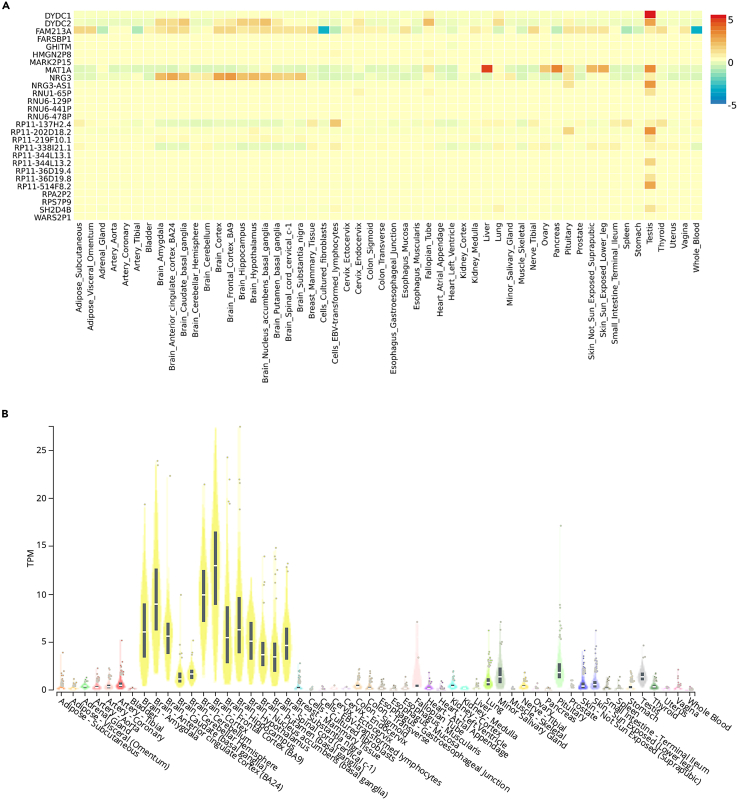
Gene expression (A) Bulk gene expression in Genotype-Tissue Expression (GTEx) v8 tissues, of mapped genes for gabapentin pain response at a genome-wide significance threshold using FUMA GENE2FUNC (average of normalized expression per label (log2 transformed)). (B**)** Bulk gene expression for *NRG3* in GTEx v8 tissues.

Functional mapping of loci was performed using FUMA, in which the pain response genomic risk locus (lead SNP rs4442490) mapped to the expression of 31 genes. A heatmap of bulk gene expression using GTEx v8 for mapped genes was produced using the FUMA GENE2FUNC tool, showing *NRG3* is expressed predominantly in central nervous system tissues (see Figure 3).

### Gene set enrichment analysis

MAGMA gene set enrichment analysis was conducted in FUMA SNP2GENE for the mapped set of 31 genes using GO terms obtained from MsigDB. A reactome pathway of the activation of SMO was implicated (p_bon_ 0.015).

The 31 mapped genes were analyzed for statistical overrepresentation in biological pathways using PANTHER Pathways and were observed to be overrepresented in the EGF receptor signaling pathway and S-adenosylmethionine biosynthesis, although these associations did not hold when a multiple testing correction was applied (see Table S2). SNPs of genome-wide significance were investigated using PhenoScanner which identified that all six SNPs of genome-wide significance were associated with DNA methylation levels of NRG3 in whole blood (see Table S3).

### Exploratory analysis of nominal associations

We conducted an exploratory analysis of nominal associations (*p* < 1 × 10^−5^) between genetic risk loci and gabapentin pain response and side effect burden. We identified 26 independently significant SNPs associated with pain response and 16 independently significant SNPs associated with side effect burden (see Table S4 and Table S5). These loci were associated to the expression of 79 and 21 genes (FUMA analysis), respectively, and gene expression heatmaps for the mapped genes are shown in Figure S5.

To investigate potential biological pathways involved in gabapentin response, we used PANTHER Pathways to test for the overrepresentation of the mapped genes in biological pathways. We found that the 79 genes (of which a mapped set of 54 was analyzed in PANTHER Pathways) associated with pain response were overrepresented in the Cadherin signaling pathway (*p* = 3.4 × 10^−20^) and the Wnt signaling pathway (*p* = 1.5 × 10^−17^) after applying a false discovery rate correction (see Table S6). We did not observe any statistically significant overrepresentation of the 21 genes associated with side effect burden in any biological pathways after a false discovery correction.

### Analysis of lead SNP association with brain imaging phenotypes in UK Biobank

A phenome-wide association study of the lead SNP rs4442490 within UK Biobank’s 40k dataset reveals Bonferroni-corrected significant differences across a range of metrics (see Table 4). Structural measures comprised volumetric and grey-white contrast variation in orbitofrontal regions. DTI-tractography implicated the superior longitudinal fasciculus. Resting state networks comprised several regions, dominated by connections between orbitofrontal/insula areas and posterior midline/parietal regions.

**Table 4:** Brain imaging-derived phenome-wide association study for rs4442490

Modality	Phenotype	β	p
Diffusion-weighted tractography	Weighted-mean orientation dispersion index in tract left superior longitudinal fasciculus	0.031	1.1 × 10^−4^
Resting-state fMRI	ICA100 edge 309	−0.029	2.6 × 10^−4^
	Node 9	STG, PHG, OFC, mediodorsal thalamus	
	Node 26	R posterior ITG, pre/postcentral gyri, insula, OFC	
	ICA100 edge 549	0.029	3.5 × 10^−4^
	Node 21	IFG, medial MFG/SFG, subgenual ACC, ventral striatum, hippocampus	
	Node 34	SPL, precuneus, pre/postcentral gyri	
	ICA100 edge 713	−0.028	5.6 × 10^−4^
	Node 10	MTG, fusiform gyrus, PHG, PCC	
	Node 39	Precuneus, SOG	
T1-weighted structural	Volume R amygdala – cortical nucleus	−0.027	8.5 × 10^−4^
	Grey-white contrast L medial OFC	0.026	9.4 × 10^−4^
	Volume R medial orbital sulcus (olfactory)	0.025	1.4 × 10^−3^

Resting-state fMRI ICA100 refers to a 100-dimension parcellation by independent components analysis. The partial correlation between each parcellation or “node” defines each “edge.” The dominant anatomical regions within each node are listed. ACC: Anterior cingulate cortex; IFG: Inferior frontal cortex; ITG: Inferior temporal gyrus; MFG: Middle frontal cortex; MTG: middle temporal gyrus; OFC: Orbitofrontal cortex; PCC: Posterior cingulate cortex; PHG: Parahippocampal gyrus; SFG: superior frontal cortex; SOG: Superior occipital cortex; SPL: Superior parietal lobule; STG: Superior temporal gyrus.

### Comparing gabapentin responders and non-responders according to lead SNP association using fMRI brain

Identifying GaPP2 participants with both genomic and neuroimaging data revealed two people who were classified as gabapentin responders – both of whom were rs4442490 TT homozygotes – and five gabapentin non-responders, who were all either TG or GG. Given the small amount of available data, an exploratory analysis was performed by which fMRI responses to punctate stimuli were compared between the TT responder and TG/GG non-responder groups. A full factorial model with pre- and post-treatment as a within-subjects effect, and responder status as a between-subjects effects, revealed significant activation within several brain regions, corrected for multiple comparisons across the whole brain volume (see Table 5). In all cases, the responders showed a decrease in punctate activation after treatment with gabapentin.

**Table 5:** Comparing rs4442490 TT responders and TG/GG non-responders: Brain regions showing significant fMRI activation changes in response to punctate stimuli following treatment with gabapentin

Region	MNI coordinates	Z	kE	Cluster p (FWE-corrected)	UK Biobank node (edge)
R Calcarine cortex	20 -86 10	4.56	34	0.007	–
L lateral OFC/IFG	−42 38 -14	4.54	126	<0.001	39 (713)
Lingual/calcarine cortex	0 -84 0	4.45	50	<0.001	–
R supramarginal cortex	54 -24 36	4.39	33	0.009	26 (309)
R middle/superior occipital cortex	32 -80 26	4.25	39	0.003	–
R superior parietal lobule	22 -50 52	4.19	47	0.001	34 (549)
L anterior/medial OFC	−22 48 -16	4.11	26	0.037	21 (549)
R anterior/lateral OFG	38 56 -8	4.08	25	0.045	–
R anterior MFG/frontal pole	32 52 8	3.83	63	<0.001	–

In all cases, there was a significant decrease in activation post-gabapentin in the responder group, but not the non-responder group.

**Figure 4:**
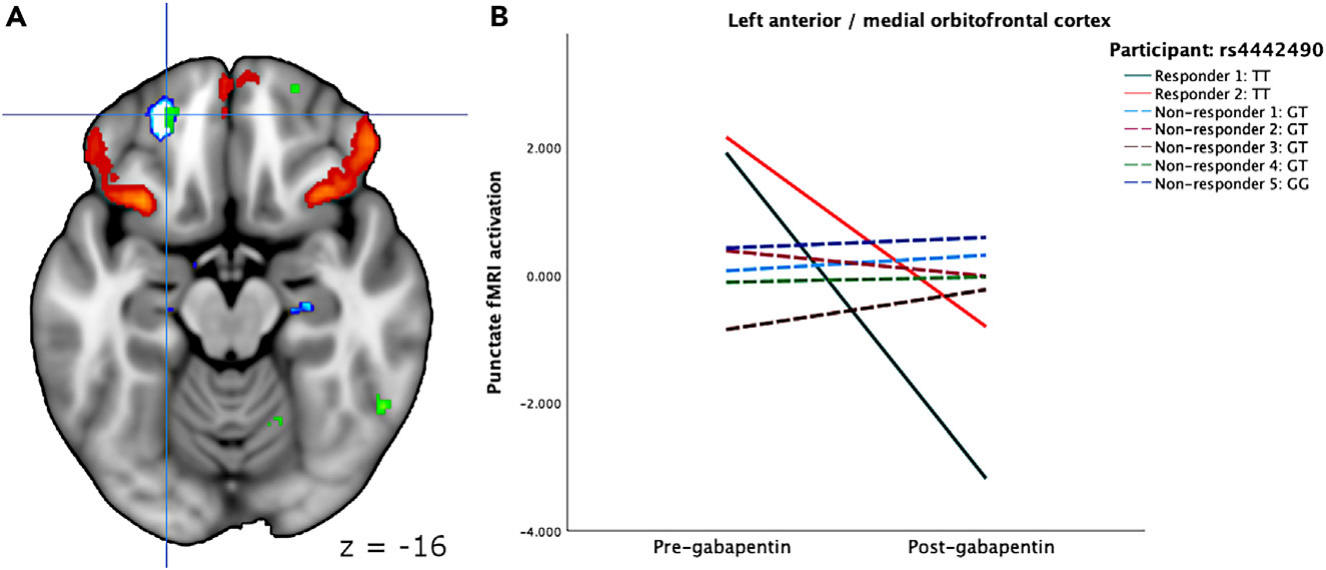
fMRI activation in response to punctate stimuli (A) Green regions denote those showing a significant whole-brain Responder X pre-post treatment interaction. To demonstrate the overlap with the UK Biobank resting-state edge 549 (node 21), the positive (red) and negative (blue) components are displayed. Crosshairs highlight MNI coordinates −22 48 -16 (left anterior/medial orbitofrontal cortex). (B) Participant-level activation for this region, demonstrating that those with the rs4442490 TT variant showed decreased activation after receiving gabapentin.

Four of the regions significantly associated with gabapentin response demonstrated an overlap with the resting state nodes associated with rs4442490, specifically within the left orbitofrontal cortex, and right posterior parietal regions. Figure 4 demonstrates the overlap between one of these regions – left anterior/medial orbitofrontal cortex – with node 21 (of edge 549), together with fMRI punctation activation for each participant.

## Discussion

We present a genome-wide association study of gabapentin response phenotypes in women with CPP and no obvious pelvic pathology, using a well-defined cohort.^9^ We identify a locus in *NRG3* associated, at a genome-wide significance threshold, with the analgesic efficacy of gabapentin in women with CPP. Our findings suggest that *NRG3*, which is predominantly expressed in the CNS, may play a neurobiologically plausible role in the response to gabapentin treatment in women with CPP.^22^

*NRG3* is one of the four genes (alongside *NRG1*, *2,* and *4*) that comprise the Neuregulin family, which code for a group of epidermal growth factor (EGFR)-like signaling proteins of the same name involved in cell–cell communication.^23^^,^^24^ The Neuregulins signal through ErbB3/4 tyrosine kinase receptors and have been shown to play a critical regulatory role in the development, maintenance, and repair of the developing and mature nervous system.^25^^,^^26^^,^^27^ *NRG3*, first discovered in 1997, has the greatest degree of CNS specificity among the Neuregulins.^23^^,^^28^ NRG3’s EGF-like domain binds specifically to the tyrosine protein kinase receptor ErbB4, enriched in the neuronal post-synaptic densities of inhibitory interneurons in the CNS.^23^^,^^28^^,^^29^ NRG3 is involved in regulating multiple biological processes within the nervous system, including interneuron migration^30^ and neurite outgrowth,^31^ as well as promoting the survival of neurons and oligodendrocytes.^32^ Further, NRG3 plays a role in glutamatergic transmission,^33^ and in the formation and maturation of synapses onto GABAergic interneurons expressing the ErbB4 receptor.^34^^,^^35^^,^^36^

Many groups have demonstrated that genetic variants in the *NRG3* gene are implicated in a range of neurodevelopmental and psychiatric disorders, including developmental delay, cognitive impairment, attentional deficits, and psychotic disorders such as schizophrenia.^37^^,^^38^^,^^39^^,^^40^^,^^41^ Additionally, recent evidence shows common genetic variants link chronic pain phenotypes and many neuroimaging traits.^42^ One schizophrenia risk-associated polymorphism (rs10748842, an intron variant) in *NRG3* was demonstrated through fMRI to impact prefrontal cortical physiology and was associated with elevated *NRG3* gene expression in the adult and fetal prefrontal cortex.^38^^,^^43^ Additionally, SNPs in *NRG3* have been linked to nicotine addiction and smoking cessation success in humans, translated from mice studies showing ErbB4 to play a role in anxiety-like behaviors during nicotine withdrawal.^44^^,^^45^ In mice, nicotine withdrawal was associated with the altered synaptic expression of ventral hippocampal NRG3 and ErbB4.^46^ Genetic alteration of ventral hippocampus ErbB4 led to an elimination of nicotine withdrawal-related anxiety-like behaviors. Mechanistically, this effect involved the alteration of GABAergic modulation of pyramidal cell activity due to the disruption of NRG3-ErbB4 signaling in the ventral hippocampus. Here we find support for the neurobiological impact of *NRG3* in the context of gabapentin efficacy in treating chronic pelvic pain. Within a large sample of UK Biobank participants, we find phenome-wide significant brain imaging features associated with rs4442490, particularly implicating prefrontal regions such as the orbitofrontal cortex. Within our very small sample of GaPP2 participants who also underwent brain imaging, we found that those who responded to gabapentin (all of whom possessed the TT variant) also showed a significant drop in left orbitofrontal activation in response to punctate stimuli, which was not seen in non-responders. Prefrontal brain regions, such as the orbitofrontal cortex, play an important role in acute and chronic pain perception.^47^ Recently, intracranial orbitofrontal cortex signals have shown as objective biomarkers able to predict chronic pain states.^48^

Many epidemiological studies have shown that genetic variation contributes to chronic pain susceptibility in a variety of chronic pain phenotypes including CPP.^49^^,^^50^^,^^51^ Further, evidence exists to explain the relationship between neuregulin signaling and chronic neuropathic pain. *NRG1*, a paralog of *NRG3* and the most studied of the Neuregulins, has been shown as a modulator of neuroinflammation in response to a multitude of CNS insults including traumatic injury that can drive neuropathic pain.^52^^,^^53^ Elevated expression levels of microRNAs (miR-155-5p) targeting *NRG3* were observed among individuals with trigeminal neuralgia, another chronic neuropathic pain condition, compared to healthy controls, suggesting possible microRNA involvement in chronic neuropathic pain development.^54^^,^^55^ Interestingly, one group has shown through early uncontrolled case series, the potential efficacy of drugs that inhibit EGFR/ErbB signaling (e.g., panitumumab) in treating neuropathic pain, calling for further assessment via clinical trials.^56^^,^^57^ Many drugs which could inhibit NRG3-ErbB4 signaling specifically already exist, although usage is limited to cancer treatment.^58^ The evidence linking genetic variants in *NRG3* (and its involvement with subsequent signaling) with neuropsychiatric phenotypes and CNS physiology, suggests biological plausibility that *NRG3* polymorphisms may play a role in gabapentin pain response. Herein we identify genetic variants associated with the analgesic efficiency of gabapentin among women with chronic pelvic pain, yet identified polymorphisms may plausibly have roles in chronic or neuropathic pain susceptibility more broadly, rather than relating specifically to gabapentin pharmacology. To our knowledge, *NRG3* has not previously been shown to be directly involved in gabapentin’s predominant mechanism of action (inhibition of voltage-gated calcium channels) or its pharmacodynamics or pharmacokinetics, nor did we identify any SNPs associated with a response within candidate genes selected due to their established involvement with gabapentin pharmacology.^59^

The present study is limited by the use of a very small sample size compared to typical non-pharmacogenomics GWAS, limiting our ability to detect smaller effect sizes. It is worth noting that smaller sample sizes can yield meaningful findings in pharmacogenomic GWAS compared with traditional GWAS of diseased cases versus healthy controls. A recent pharmacogenomics review published in 2021 reported a median sample size of 738 and 669 for genome-wide association studies investigating drug response and adverse drug reactions, respectively.^16^ Additionally, pharmacogenomic GWAS typically report larger effect sizes, a finding that is not explained by their smaller sample sizes and may be related to narrow, well-defined phenotypes, typically relating to clearly defined biological pathways that typically lack genetic heterogeneity.^60^ Therefore, while our sample size remains small, we remain confident in our findings at a genome-wide significance threshold due to the large effect size of the lead SNPs, the biological plausibility of the role of *NRG3*, the deeply and narrowly phenotyped cohort of women included in this study, and the robust data collection facilitated by a large clinical trial.

Our findings regarding the overrepresentation of mapped genes in the Cadherin and Wnt signaling pathways, which are known to be involved in neuropathic pain and pain processing,^61^^,^^62^ should be interpreted with caution given the nominal association threshold that genes within them are derived from. Nonetheless, these findings provide a promising starting point for future investigations into the biological mechanisms underlying variability in gabapentin response.

Successful replication or validation would provide stronger evidence to support the biological involvement of *NRG3* and pave the way for the potential utility of an SNP-based test to predict drug response and stratify individuals likely to benefit from gabapentin treatment. Our lead *NRG3* SNP (rs4442490) has a high population minor allele frequency (MAF = 0·43 in TOPMED), making such a test plausible. Such a test could be of considerable benefit, as women in our gabapentin pain responder group experienced an average 47% reduction in average pain, which corresponds to a patient-reported outcome of “*very much improved.*”^10^ Given the unfavorable side effect profile of gabapentin and the risk of dependence, limiting its use in patients where it may have a limited therapeutic effect is highly desirable.

Given the predominately European population included in the GaPP2 trial, our findings are primarily applicable to individuals of European ancestry. Although the population MAF of rs4442490 remains common across ethnicities, it varies significantly (MAF 0.22–0.49). Hence, the utility of a genotypic test in non-European populations remains unclear, necessitating further investigation. Several methods of replication and validation are plausible. For instance, *in vitro* studies involving NRG3 knockout mice could potentially elucidate the role and potential causality of rs4442490. Analysis of genetic data collected as part of randomized controlled trials of gabapentin for other pain conditions remains a possibility, although the inclusion of genetic profiling in trials remains infrequent. We strongly encourage investigators to consider the inclusion of genetic profiling in future randomized controlled trials to enable secondary analysis such as that presented herein, making optimal use of their rich and well-characterised datasets. Ultimately, we believe prospective validation in a randomized controlled trial of gabapentin for the treatment of CPP that utilizes an SNP-based test as an inclusion criterion is likely necessary prior to the development of an SNP-based test as a decision support tool for routine use that could aid in a decision to treat CPP with gabapentin.

In summary, we have identified genetic variants within the *NRG3* gene associated with the analgesic efficacy of gabapentin in women with chronic pelvic pain. Our findings could contribute to the development of more effective and individualized pain management strategies. However, further research is needed to validate these findings and explain the biological pathways involved to understand the complex interplay between genetics, gabapentin, and pain response variability.

### Limitations of the study

This study is limited by a small sample size, which reduces our ability to detect smaller effect sizes. We advise caution in the interpretation of findings that are derived from genetic associations identified at a nominal association threshold. Given our study cohort, the results are primarily applicable to individuals of European ancestry, and extrapolation to non-European populations should be approached with caution. Additionally, the utility of our findings would benefit from replication and validation, such as through *in vitro* studies or genetic data analysis from other gabapentin trials.

## STAR★Methods

### Key resources table

**Table:** 

REAGENT or RESOURCE	SOURCE	IDENTIFIER
**Deposited data**		

GWAS summary statistics: Gabapentin pain response	NHGRI-EBI GWAS Catalog	GCST90428068
GWAS summary statistics: Gabapentin side-effect burden	NHGRI-EBI GWAS Catalog	GCST90428069

**Critical commercial assays**		

Oragene OG-500 saliva collection kits	DNA Genotek, Ontario, Canada	https://www.dnagenotek.com/row/index.html (OG-500)
prepIT.L2P	DNA Genotek, Ontario, Canada	https://www.dnagenotek.com/row/products/reagents-preparation/prepIT/PT-L2P.html
Quant-iT PicoGreen dsDNA Assay Kits	Thermo Fisher Scientific Inc., MA, USA	https://www.thermofisher.com/order/catalog/product/P11496
NanoDrop Spectrophotometer	Thermo Fisher Scientific Inc., MA, USA	https://www.thermofisher.com/uk/en/home/industrial/spectroscopy-elemental-isotope-analysis/molecular-spectroscopy/uv-vis-spectrophotometry/instruments/nanodrop/instruments/nanodro-one.html
Illumina Global Screening Array/Illumina iScan	Illumina Inc., San Diego, USA	N/A
MAGNETOM Verio 3T scanner	Siemens Medical Solutions, Malvern, USA	https://www.siemens-healthineers.com/en-us/magnetic-resonance-imaging/3t-mri-scanner/magnetom-verio

**Software and algorithms**		

GenomeStudio (v2·0·3)	Illumina Inc., San Diego, USA	https://emea.illumina.com/techniques/microarrays/array-data-analysis-experimental-design/genomestudio.html
GenCall (v6·3·0)	Illumina Inc., San Diego, USA	N/A
PLINK v1·07	Purcell et al.^63^	https://www.cog-genomics.org/plink/
SHAPEIT	Delaneau et al.^64^	https://mathgen.stats.ox.ac.uk/genetics_software/shapeit/shapeit.html
Michigan Imputation Server	N/A	https://imputationserver.sph.umich.edu
SNPTEST v2·5·4-beta1	Marchini et al.^65^	https://www.chg.ox.ac.uk/∼gav/snptest/
STRING (v11·5)	Szklarczyk et al.^66^	https://string-db.org/
LocusZoom	Pruim et al.^67^	http://locuszoom.org/
FUMA v1·5·2	Watanabe et al.^68^	https://fuma.ctglab.nl/
Genotype-Tissue Expression (GTEx) v8	GTEx Consortium^69^	https://gtexportal.org/home/
PANTHER (v17·0) Pathways	Mi et al.^70^	https://www.pantherdb.org/pathway/
Oxford Brain Imaging Genetic (BIG40) web server	Smith et al.^71^	https://open.win.ox.ac.uk/ukbiobank/big40/
PheWeb	Gagliano et al.^72^	https://open.win.ox.ac.uk/ukbiobank/big40/pheweb33k/
easyROC	Goksuluk et al.^17^	N/A
SPM12	Functional Imaging Laboratory, UCL Queen Square Institute of Neurology, London, UK	https://www.fil.ion.ucl.ac.uk/spm/software/spm12/

### Resource availability

#### Lead contact

Further information and requests for resources should be directed to the lead contact, Lucy HR Whitaker (lucy.whitaker@ed.ac.uk).

#### Materials availability

This study did not generate new unique reagents.

#### Data and code availability

• Genome-wide association summary statistics have been deposited at the NHGRI-EBI Catalog of human genome-wide association studies and are publicly available as of the date of publication. Accession numbers are listed in the key resources table.
• This paper does not report original code. Our analyses used publicly available software which are cited in the methods.
• Any additional information required to reanalyse the data reported in this work paper is available from the lead contact upon request. Participant data in this study is derived from the GaPP2 trial. Please see Horne et al. (2020)^9^ for contact details for data requests.

### Experimental model and study participant details

#### Study participants

Women with CPP and no pelvic pathology who received gabapentin as part of the UK-wide multicentre randomised placebo-controlled trial, GaPP2, were eligible for inclusion in this study (*n* = 153).^9^ The GaPP2 trial protocol and results with full details of the inclusion and exclusion criteria, participants and dosing regimen are published elsewhere.^9^^,^^73^ In summary, eligible participants for the GaPP2 trial were women aged 18–50 years with chronic pelvic pain lasting at least three months (with or without dysmenorrhoea and/or dyspareunia), and no obvious pelvic pathology at laparoscopy (e.g., macroscopic endometriosis lesions, complex ovarian cysts or ovarian cysts of >5 cm, fibroids of >3 cm, or dense adhesions). Laparoscopy was performed at least two weeks and less than 36 months prior to consent. Participants were recruited from gynecology clinics, CPP clinics, gynecology wards and day surgery units. Pelvic pain was defined as pain located within the true pelvis (between and below the anterior iliac crests). CPP was defined using the Royal College of Obstetricians and Gynecologists 2012 Green-top guideline definition and the 2012 International Association for the Study of Pain taxonomy.^74^^,^^75^ A pre-requisite for randomisation into the GaPP2 trial was that participants had to have at least 2 out of 4 of their ‘worst pelvic pain’ scores a score of 4 or higher on a 0–10 numerical rating score over the preceding four weeks (captured using a bespoke text messaging system). Participants received gabapentin for a period of 16 weeks. The dosing regimen included a 4-week titration phase where participants increased their gabapentin until they perceived they were gaining adequate analgesia or side effects prevented further dose increases, to a maximum dose of 2700 mg daily. Within the study the term woman equates to female sex rather than gender identity, owing to recognised sex-based differences in pain thresholds, sensitivity and underlying neuroimmune modulation.^76^ Ethical approval for the GaPP2 trial was obtained from the UK Coventry and Warwick Research Ethics Committee (REC 15/WM/0036). For inclusion in this study, participants were able to optionally provide a saliva sample at entry to the GaPP2 trial. Saliva samples were collected using Oragene OG-500 (DNA Genotek, Ontario, Canada) saliva collection kits following manufacturer’s instructions.

### Method details

#### Defining gabapentin response phenotypes

Phenotype definition for genome-wide association described gabapentin response through categorisation of pain response (i.e., analgesic effect) and side effect burden (i.e., degree of intolerance). We aimed to capture the analgesic effect of gabapentin by categorizing cases into pain responders and non-responders. Pain response was defined using the coprimary outcomes of the GaPP2 trial which were worst and average pain scores collected at 13–16 weeks post-randomisation reported by participants on a numerical rating scale (0–10). To identify gabapentin pain responders, we applied a categorical definition of a ≥30% relative reduction in worst and/or average pain scores after gabapentin exposure. This threshold was based on an IMMPACT consensus statement,^10^ which stated that a ≥30% reduction in pain intensity corresponds to a “*moderately important*” improvement in pain. A density plot of worst and average pain response is available in Figure S1.

To categorize gabapentin side effect burden, we used a categorical threshold of ≥3 side effects reported during the study period. We adopted this approach due to the typically mild and high frequency nature of side effects experienced within the GaPP2 trial.^9^ Additionally, due to our small sample size we opted to use a binary outcome and therefore cases were categorized into those reporting ≥3 side effects and those reporting <3 side effects for subsequent analysis.

#### DNA extraction and genotyping

DNA was extracted from 500ul of saliva using prepIT.L2P (DNA Genotek, Ontario, Canada) following manufacturer’s instructions. DNA quality was assessed using a Quant-iT PicoGreen dsDNA Assay Kits (Thermo Fisher Scientific Inc., MA, USA) and a NanoDrop Spectrophotometer (Thermo Fisher Scientific Inc., MA, USA). Samples were genotyped at Edinburgh Clinical Research Facility using an Illumina Global Screening Array on an Illumina iScan (Illumina Inc., San Diego, USA). Genotypes were called using GenomeStudio (v2·0·3) and genotypes with a GenCall (v6·3·0) cut-off of <0·15 were declared as missing.

#### MRI scanning

Within the sample taking part in the GaPP2 trial, a small number also took part in a mechanistic fMRI study, undergoing MRI brain scans before and after 12 weeks of treatment. Baseline scans took place between January 2016 and November 2018. Scanning took place at the Edinburgh Imaging Facility QMRI,^77^ using a 3T Siemens MAGNETOM Verio scanner. Scanning comprised of a high-resolution structural scan; functional imaging during both a resting state scan, and the application of punctate stimuli; field map acquisitions; and pseudo-continuous arterial spin labeling. This paper will focus on the functional responses to punctate stimuli, and resting state data.

BOLD functional images were acquired using an echoplanar T2∗ GRAPPA gradient echo pulse sequence, with TR 2500ms, TE 30ms, flip angle 90′, FOV 192mm, 45 interleaved contiguous slices, and resolution 3 × 3 × 3mm. A T1-weighted structural image was acquired using an MPRAGE sequence, with TR 2300ms, TE 2.98ms, FoV 256mm, resolution 1 × 1 × 1mm, and flip angle 9’. The punctate task was 10 min 50 s, i.e., 260 volumes, the first four of which were discarded to reduce T1 saturation effects. The resting state scan lasted 8 min 20 s, with 200 volumes. The resting state scan was performed prior to the punctate scan to avoid potentially exacerbating pain during resting state. Gradient field maps were acquired with the same dimensions as the functional data, and TE1 4.92ms, TE2 7.38ms. The pre-treatment scan occurred at visit 1 of the trial schedule, and the post-treatment scan between weeks 13 and 16 (visit 5 of the trial schedule). Both scans were identical, and phase-locked to the stage of menstrual cycle for each participant.

#### Punctate experiment

The punctate scan involved the application of 39 punctate stimuli, using a 300 g Touch Test von Frey filament (6.65mm), within an event-related design, directed by timed auditory cues. Mean interstimulus interval = 16s, random jitter = ±2.5s. Stimuli were applied to the lower abdomen, 10cm above the superior edge of the pubic bone.

### Quantification and statistical analysis

#### Quality control and imputation

Quality control and imputation was performed to ensure the accuracy and completeness of the genetic data.^78^ PLINK v1·07^63^ was used to exclude samples with discordant sex information, outlying missingness (≥5%), and heterozygosity (>2 standard deviations from the mean). Pairwise identity-by-descent (IBD) was analyzed and one individual from each related pair was excluded (pi-hat >0·185). Principal components analysis was conducted and, given the preponderance of European ancestry individuals in the GaPP2 trial, non-European ancestry individuals were excluded. For variant quality control, the following filters were applied: SNPs with genotypic call rates ≤95%, Harvey-Weinberg equilibrium *p* ≤ 0·00001, and minor allele frequency ≤5% were excluded. A strict (≤5%) minor allele frequency cut-off was adopted due to the small sample size in this study. To impute missing genotypes, genotypes were pre-phased using SHAPEIT^64^ and the Haplotype Reference Consortium (HRC) reference panel was used via the Michigan Imputation Server.^79^ After imputation, post-imputation variant quality control was performed by excluding SNPs with poor imputation quality (information score ≤0.4) and minor allele frequency ≤5%.

#### Genome-wide association analyses

Genome-wide association analyses were conducted to investigate the relationship between two phenotypes of interest: gabapentin pain response and gabapentin side effect burden. SNPTEST v2·5·4-beta1^65^ was used and a frequentist additive logistic regression model without any covariates to autosomes and chromosome X was applied. We did not include covariates in our genome-wide association analysis due to a lack of significant evidence linking them to our phenotypes of interest and to avoid diminishing the statistical power of our study given our limited sample size. To determine statistical significance, two thresholds were adopted: a genome-wide significance threshold of *p* < 5 × 10^−8^ and a nominal significance threshold of *p* < 1 × 10^−5^. SNPs that reached the genome-wide significance threshold were considered to be associated with the phenotypes of interest, while SNPs that reached the nominal significance threshold were considered suggestive and deserving of further investigation.

#### Candidate gene association analysis

Candidate gene analysis was performed for selected genes due to either their relevance to gabapentin pharmacology or though STRING (v11·5) network analysis with any gene identified at a genome-wide significance threshold.^66^ For each candidate gene, an association analysis to identify any significant SNPs was conducted. We considered all SNPs within the candidate gene plus 500kb upstream and downstream regions. To control for multiple testing, we used a Bonferroni correction and considered a *p*-value of less than 0·05/number of tests as significant.

#### Functional mapping and pathway analysis

Independently associated SNPs were identified using r^2^ ≥ 0·6. Regional association plots for loci of interest were produced using LocusZoom.^67^ Loci were assigned to mapped genes positionally and through expression quantitative trait loci (eQTL) and chromatin integration using SNP2GENE in FUMA v1·5·2.^68^ The FUMA GENE2FUNC tool was used to create bulk gene expression heatmaps using Genotype-Tissue Expression (GTEx) v8.^69^ Identified gene sets were tested for statistical overrepresentation in biological pathways using PANTHER (v17·0) Pathways.^70^

#### Analysis of lead SNPs association with brain imaging phenotypes in UK Biobank

To provide more insight into potential central neurological pathways associated with the genetic signatures of gabapentin response, we looked up genome-wide significant loci lead SNPs in phenome-wide association study brain imaging-derived phenotypes within the UK Biobank.^80^ Brain imaging phenotypes were provided by the Oxford Brain Imaging Genetic (BIG40) web server^71^ and examined using the PheWeb interface.^72^ BIG40 is derived from the 40k release of UK Biobank, using data from 33,224 people, of whom 17,411 are genetic females, with mean age 64·4 years (7.5 SD). Imaging pre-processing pipelines have previously been described in detail.^81^^,^^82^ Derived phenotypes include regional measures of structural volume, cortical thickness, cortical grey-white contrast; white matter tract mean orientation and orientation dispersion; and resting-state fMRI node amplitude and inter-node correlation. Phenotypes achieving Bonferroni-corrected significance of *p* < 0·05 across the 3935 imaging phenotypes were reported.

#### Punctate fMRI data preprocessing and analysis

The punctate fMRI data were analyzed using SPM12. Data were un-warped using the field maps, and realigned to a mean image, which was co-registered to each person’s T1-weighted structural image. This was in turn segmented and warped to the MNI space, with warp parameters applied to the co-registered functional images. The data were finally smoothed with a 5mm FWHM kernel. Quality assurance procedures examined the data for slice spikes and significant motion (defined as a single volume motion exceeding half a voxel i.e., 1.5mm). No participants demonstrated motion issues. For each participant, the onset of each punctate stimulus was represented as a delta function, and convolved with the canonical haemodynamic response function, as well as its temporal and dispersion derivatives. First level design matrices modeled the pre- and post-treatment scans, and nuisance regressors representing motion parameters were included.

#### Comparing gabapentin responders and non-responders according to lead SNP association using fMRI

For those for whom both genetic and imaging data was available, the gabapentin pre- and post-treatment scans were compared. The first-level contrast of post-treatment > pre-treatment punctate vs. rest was taken into a random effects analysis comparing SNP variants, with clusters achieving a whole-brain, FWE-corrected significance of *p* < 0·05 being reported.

### Additional resources

This study uses data derived from the GaPP2 trial^9^^,^^73^ (ISRCTN registry, ISCRTN77451762).
