# Supplementary material for: Genome-wide association reveals a locus in neuregulin 3 associated with gabapentin efficacy in women with chronic pelvic pain
Source: iScience. 2024 Jul 15;27(8):110370. doi: 10.1016/j.isci.2024.110370 (PMC11384074; doi:10.1016/j.isci.2024.110370)
Supplement: Document S1. Figures S1–S5 and Tables S1–S6 [file mmc1.pdf]

**iScience, Volume *27***

## Supplemental information

# Genome-wide association reveals a locus in neuregulin 3 associated with gabapentin efficacy in women with chronic pelvic pain

**Scott C. Mackenzie, Nilufer Rahmioglu, Liana Romaniuk, Frances Collins, Lydia Coxon, Heather C. Whalley, Katy Vincent, Krina T. Zondervan, Andrew W. Horne, and Lucy H.R. Whitaker**

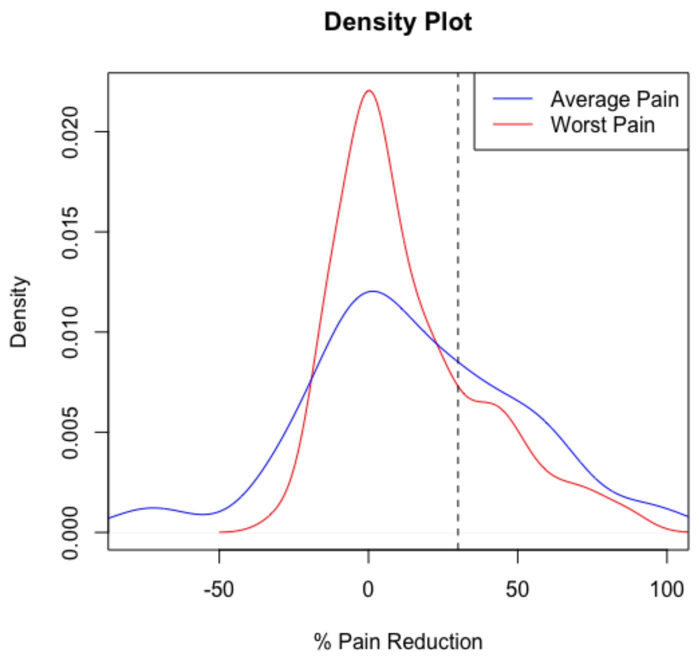


**Figure S1. Density plot of gabapentin pain response from GaPP2 trial participants included in GWAS analysis. Related to STAR Methods.**

Density plot of gabapentin pain response from GaPP2 trial participants included in subsequent GWAS analysis (n=71) for average and worst percentage reduction in pain scores. Vertical dashed line represents a 30% reduction in pain, representing a representing a "*moderate improvement*".

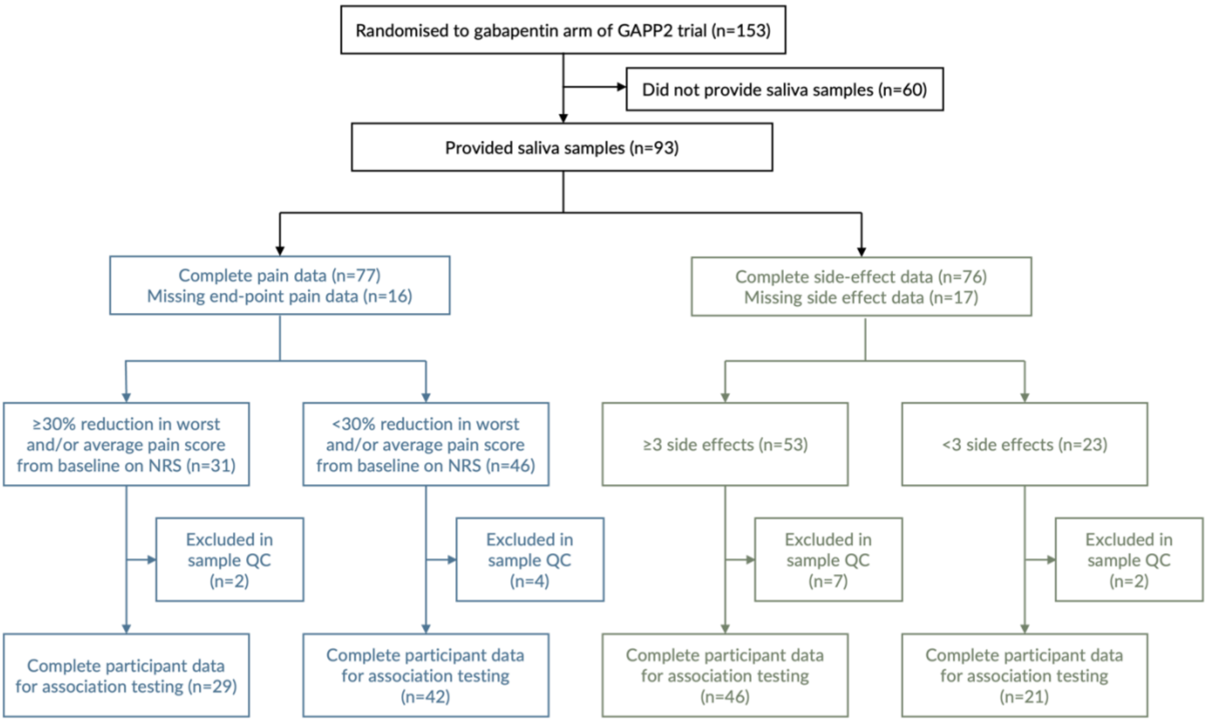


**Figure S2. Consort diagram of study participants from GaPP2 trial included in GWAS study. Related to Tables 1-2.**

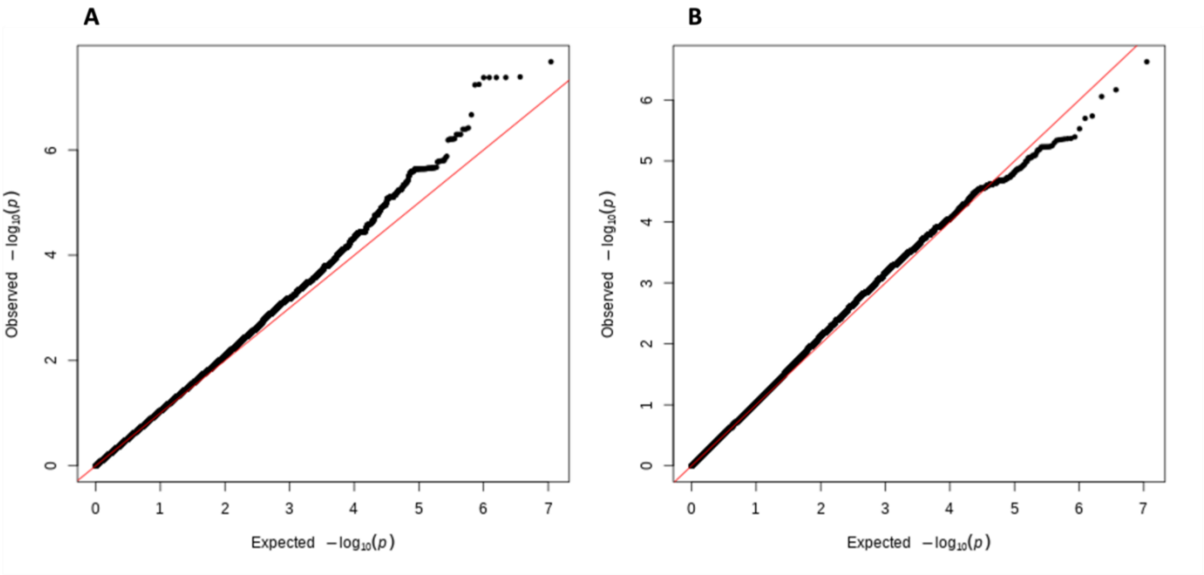


**Figure S3. QQ plots of gabapentin response and side-effects. Related to Figure 1.**
A) Gabapentin pain response (λ = 1.046); B) Gabapentin side-effect burden (λ = 1.132).

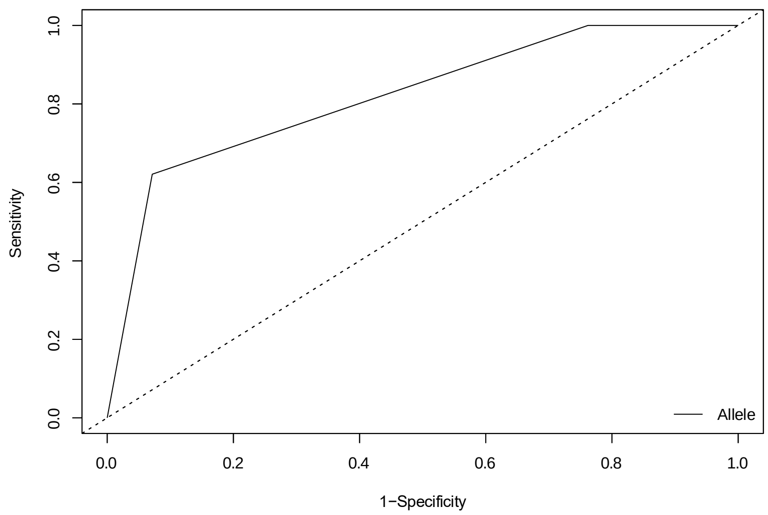


**Figure S4. Receiver operating characteristic (ROC) curve of genotypic test for rs4442490 at predicting gabapentin pain response. Related to Figure 1.**
Area under curve 0.82 (95% CI 0.74–0.90).

**A**

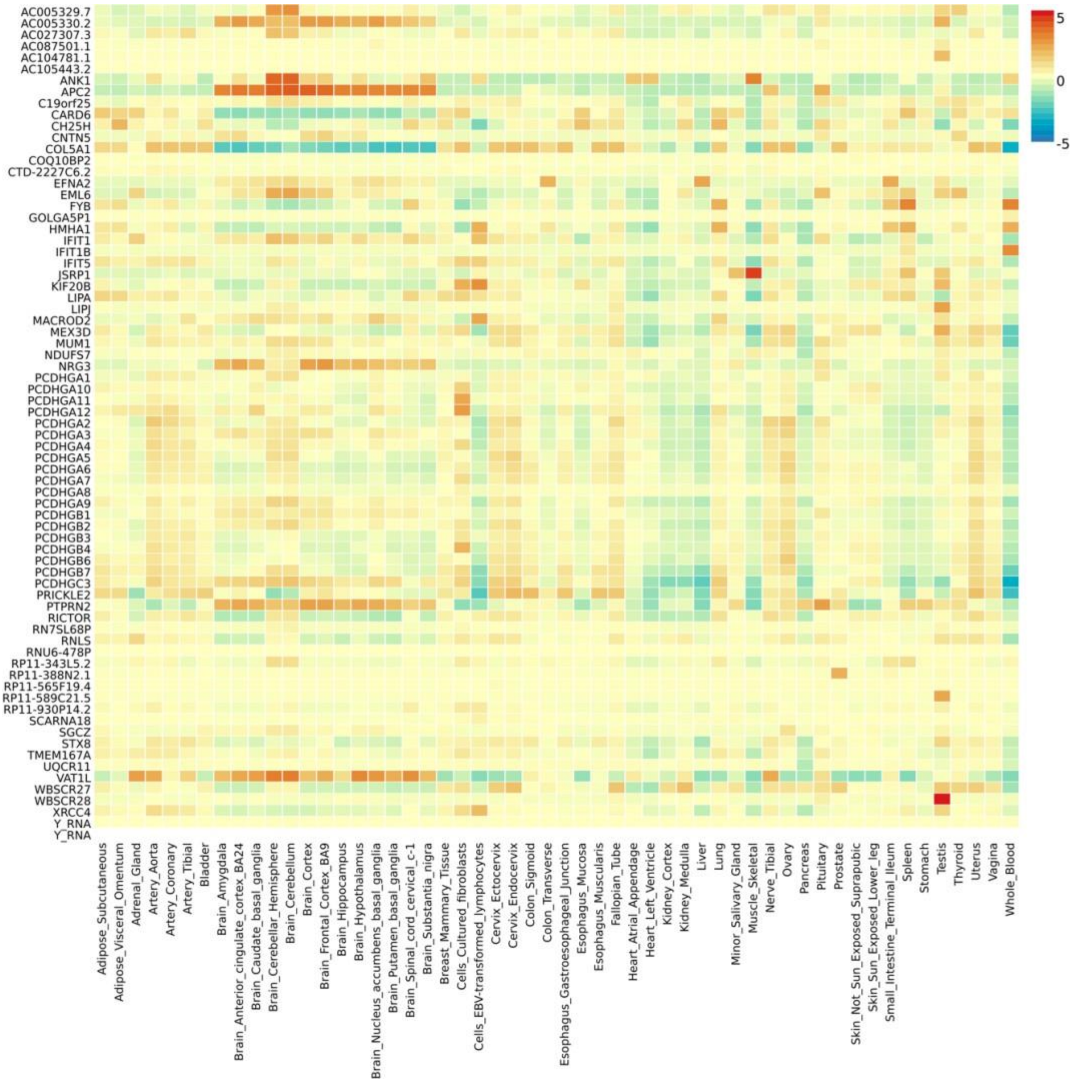


**B**

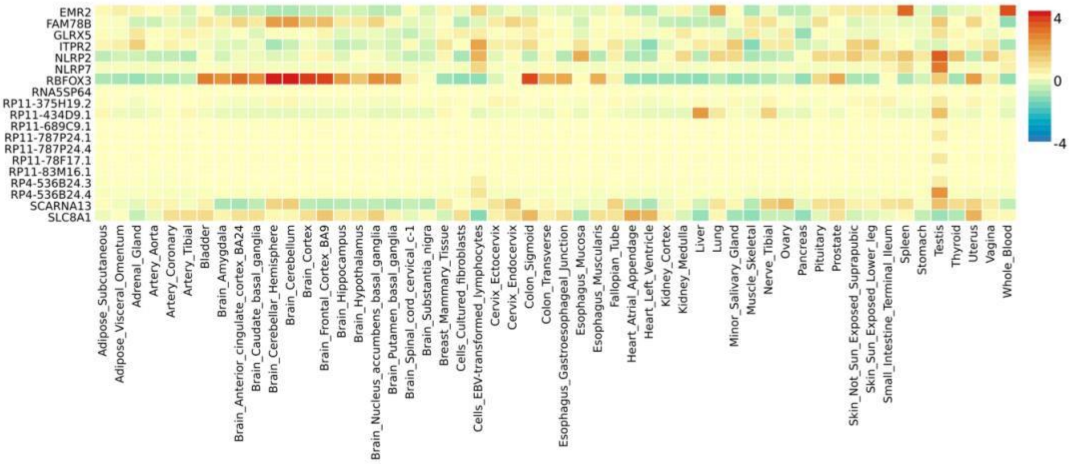


**Figure S5. Tissues of mapped genes for gabapentin pain response and side-effect burden. Related to Tables S4-S6.**

Bulk gene expression in Genotype-Tissue Expression (GTEx) v8 tissues of mapped genes for A: gabapentin pain response and B: side-effect burden at a nominal significance threshold using FUMA GENE2FUNC (average of normalised expression per label (log2 transformed)).

| SNP (rsID) | Genotype | Genotype Distribution (n) | |
|---|---|---|---|
| | | Gabapentin pain responders (n=29) | Gabapentin pain non-responders (n=42) |
| rs4442490 | GG | 0 | 10 |
| | TG | 11 | 29 |
| | TT | 18 | 3 |
| rs4933859 | CC | 0 | 9 |
| | TC | 6 | 25 |
| | TT | 23 | 8 |
| rs10885506 | GG | 0 | 9 |
| | TG | 6 | 25 |
| | TT | 23 | 8 |
| rs1923561 | GG | 0 | 9 |
| | AG | 6 | 25 |
| | AA | 23 | 8 |
| rs2026495 | CC | 0 | 9 |
| | AC | 6 | 25 |
| | AA | 23 | 8 |
| rs1923565 | GG | 0 | 9 |
| | GC | 6 | 25 |
| | CC | 23 | 8 |

**Table S1. Genotype distribution for SNPs of genome-wide significance for gabapentin pain response. Related to Figure 1, Table 3.**

| | | | | | | |
|---|---|---|---|---|---|---|
| Analysis Type: | PANTHER Overrepresentation Test (Released 20221013) | | | | | |
| Annotation Version and Release Date: | PANTHER version 17·0 Released 2022-02-22 | | | | | |
| Analyzed List: | Client Text Box Input (Homo sapiens) | | | | | |
| Reference List: | Homo sapiens (all genes in database) | | | | | |
| Test Type: | FISHER | | | | | |
| Correction: | NONE | | | | | |
| PANTHER Pathways | Homo sapiens - REFLIST (20589) | Client Text Box Input (7) | Client Text Box Input (expected) | Client Text Box Input (over/under) | Client Text Box Input (fold Enrichment) | Client Text Box Input (raw P-value) |
| S-adenosylmethionine biosynthesis (P02773) | 4 | 1 | 0 | + | > 100 | 1·70E-03 |
| EGF receptor signaling pathway (P00018) | 142 | 1 | 0·05 | + | 20·71 | 4·76E-02 |

**Table S2. PANTHER Pathway statistical overrepresentation analysis of genes mapped gabapentin pain response at a genome-wide significance threshold. Related to Figure 3.**

Results of PANTHER Pathway statistical overrepresentation analysis of genes mapped gabapentin pain response at a genome-wide significance threshold. 7 protein-coding genes mapped and 24 genes unrecognised for gabapentin pain response.

| rsid | a1 | a2 | trait | efo | study | pmid | ancestry | year | tissue | marker | location | p | direction | n | dataset |
|---|---|---|---|---|---|---|---|---|---|---|---|---|---|---|---|
| rs4442490 | G | T | DNA methylation | GO_0006306 | BIOSQTL | 27918535 | European | 2017 | Whole blood | cg25655578 | chr10:84649078 | 1.43E-20 | - | 3841 | BIOSQTL_methQTL_EUR_2017 |
| rs4442490 | G | T | DNA methylation | GO_0006306 | BIOSQTL | 27918535 | European | 2017 | Whole blood | cg16197188 | chr10:84559444 | 2.47E-20 | + | 3841 | BIOSQTL_methQTL_EUR_2017 |
| rs4442490 | G | T | DNA methylation | GO_0006306 | BIOSQTL | 27918535 | European | 2017 | Whole blood | cg06106653 | chr10:84719854 | 4.46E-06 | + | 3841 | BIOSQTL_methQTL_EUR_2017 |
| rs2026495 | C | A | DNA methylation | GO_0006306 | BIOSQTL | 27918535 | European | 2017 | Whole blood | cg25655578 | chr10:84649078 | 6.51E-12 | - | 3841 | BIOSQTL_methQTL_EUR_2017 |
| rs2026495 | C | A | DNA methylation | GO_0006306 | BIOSQTL | 27918535 | European | 2017 | Whole blood | cg16197188 | chr10:84559444 | 3.46E-06 | + | 3841 | BIOSQTL_methQTL_EUR_2017 |
| rs1923561 | G | A | DNA methylation | GO_0006306 | BIOSQTL | 27918535 | European | 2017 | Whole blood | cg25655578 | chr10:84649078 | 5.38E-12 | - | 3841 | BIOSQTL_methQTL_EUR_2017 |
| rs1923561 | G | A | DNA methylation | GO_0006306 | BIOSQTL | 27918535 | European | 2017 | Whole blood | cg16197188 | chr10:84559444 | 2.44E-06 | + | 3841 | BIOSQTL_methQTL_EUR_2017 |
| rs10885506 | G | T | DNA methylation | GO_0006306 | BIOSQTL | 27918535 | European | 2017 | Whole blood | cg25655578 | chr10:84649078 | 9.14E-11 | - | 3841 | BIOSQTL_methQTL_EUR_2017 |
| rs10885506 | G | T | DNA methylation | GO_0006306 | BIOSQTL | 27918535 | European | 2017 | Whole blood | cg16197188 | chr10:84559444 | 3.84E-06 | + | 3841 | BIOSQTL_methQTL_EUR_2017 |
| rs4933859 | C | T | DNA methylation | GO_0006306 | BIOSQTL | 27918535 | European | 2017 | Whole blood | cg25655578 | chr10:84649078 | 6.51E-12 | - | 3841 | BIOSQTL_methQTL_EUR_2017 |
| rs4933859 | C | T | DNA methylation | GO_0006306 | BIOSQTL | 27918535 | European | 2017 | Whole blood | cg16197188 | chr10:84559444 | 3.01E-06 | + | 3841 | BIOSQTL_methQTL_EUR_2017 |
| rs493385 | C | T | DNA methylation | GO_0006306 | Gaunt T | 27036880 | European | 2016 | Whole blood | cg00699941 | chr2:86332848 | 7.31E-08 | - | 837 | Gaunt-T_methQTL-Antenatal_EUR_2016 |

**Table S3. PhenoScanner epigenetic results for SNPs of genome-wide significance. Related to Figure 3.**

| Chromosome | Lead SNP | EA | NEA | EAF | EAF in gabapentin pain responders | EAF in gabapentin pain non-responders | p | OR (95% CI) | Nearest gene | Function |
|---|---|---|---|---|---|---|---|---|---|---|
| 10 | rs4442490 | T | G | 0.58 | 0.81 | 0.42 | 2.11E-08 | 18.82 (4.86-72.83) | NRG3 | intronic |
| 5 | rs2289546 | A | T | 0.18 | 0.38 | 0.04 | 6.14E-07 | NA | FYB | intronic |
| 7 | rs34469079 | C | T | 0.90 | 1.00 | 0.83 | 1.33E-06 | NA | AC105443.2 | intergenic |
| 21 | rs2837310 | C | T | 0.59 | 0.76 | 0.48 | 1.42E-06 | 15.33 (3.98-58.96) | PCP4 | intergenic |
| 6 | rs78657131 | C | T | 0.93 | 1.00 | 0.87 | 1.59E-06 | NA | RP3-399J4.1 | intergenic |
| 17 | rs67802731 | G | C | 0.75 | 0.93 | 0.63 | 1.69E-06 | 15.53 (3.77-64.03) | STX8:AC087501.1 | ncRNA_intronic |
| 16 | rs4887927 | T | C | 0.85 | 0.99 | 0.76 | 2.70E-06 | NA | VAT1L | intronic |
| 8 | rs1819952 | C | T | 0.83 | 0.97 | 0.73 | 3.00E-06 | NA | ANK1 | intronic |
| 8 | rs111969942 | C | G | 0.94 | 1.00 | 0.89 | 3.60E-06 | NA | SGCZ | intronic |
| 10 | rs11597842 | C | A | 0.75 | 0.93 | 0.63 | 4.07E-06 | NA | NRG3 | intergenic |
| 9 | rs10983662 | T | G | 0.05 | 0.13 | 0.00 | 5.24E-06 | NA | RP11-388N2.1 | downstream |
| 9 | rs3128618 | G | C | 0.21 | 0.38 | 0.09 | 5.49E-06 | 10.82 (3.24-36.13) | COL5A1 | intronic |
| 7 | rs10262371 | A | G | 0.38 | 0.56 | 0.25 | 5.73E-06 | 10.01 (2.92-34.38) | WBSCR28 | intergenic |
| 7 | rs4019378 | C | T | 0.36 | 0.56 | 0.23 | 5.82E-06 | 7.81 (2.69-22.68) | PTPRN2 | intronic |
| 4 | rs6829634 | G | A | 0.55 | 0.75 | 0.41 | 6.50E-06 | 7.15 (2.61-19.59) | RNU6-931P | intergenic |
| 10 | rs7074555 | A | G | 0.06 | 0.15 | 0.00 | 6.52E-06 | NA | LIPA | intronic |
| 20 | rs429535 | T | G | 0.71 | 0.86 | 0.60 | 6.79E-06 | 14.88 (3.64-60.83) | MACROD2 | intronic |
| 5 | rs13177759 | T | C | 0.77 | 0.95 | 0.65 | 7.27E-06 | 11.16 (3-41.55) | XRCC4 | intronic |
| 5 | rs28461214 | G | A | 0.89 | 1.00 | 0.82 | 7.43E-06 | NA | PCDHGA@ | intronic |
| 16 | rs57106598 | A | C | 0.95 | 1.00 | 0.91 | 7.95E-06 | NA | U95743.1 | intergenic |
| 3 | rs7618727 | G | C | 0.92 | 1.00 | 0.86 | 7.95E-06 | NA | PRICKLE2 | intronic |
| 19 | rs3954337 | T | C | 0.91 | 1.00 | 0.85 | 7.95E-06 | NA | MUM1 | intronic |
| 13 | rs1348467 | C | T | 0.07 | 0.15 | 0.01 | 8.32E-06 | NA | AL450423.1 | intergenic |
| 11 | rs79090474 | T | C | 0.92 | 1.00 | 0.86 | 8.78E-06 | NA | CNTN5 | intronic |
| 9 | rs79679217 | T | C | 0.20 | 0.36 | 0.08 | 9.53E-06 | 9.48 (3.02-29.72) | COL5A1 | intronic |
| 2 | rs6545445 | G | C | 0.24 | 0.43 | 0.11 | 9.78E-06 | 7.23 (2.63-19.89) | EML6 | intronic |

**Table S4. Table of genomic risk loci for gabapentin pain response at a nominal association threshold ($p < 1 \times 10^{-5}$). Related to Figure S5.**

Note: OR (95% CI) is not presented where EAF in cases or controls is <0.05 or >0.95. EA: effect allele; NEA: non-effect allele; EAF: effect allele frequency; OR: odds ratio; CI: confidence interval.

| Chromosome | Lead SNP | EA | NEA | EAF | EAF in ≥3 side effects | EAF in <3 side effects | p | OR (95% CI) | Nearest gene | Function |
|---|---|---|---|---|---|---|---|---|---|---|
| 1 | rs1316805 | C | T | 0.94 | 1.00 | 0.81 | 8.75E-07 | NA | RP11-7G12.1 | intergenic |
| 17 | rs4789951 | G | A | 0.34 | 0.47 | 0.07 | 2.35E-07 | 20.64 (19.1–22.18) | RBFOX3 | intronic |
| 6 | rs9394790 | G | A | 0.86 | 0.96 | 0.65 | 1.83E-06 | NA | AL136967.1 | intergenic |
| 19 | rs11666594 | C | T | 0.14 | 0.19 | 0.02 | 2.00E-06 | NA | EMR2 | intergenic |
| 2 | rs1605857 | T | C | 0.86 | 0.92 | 0.73 | 2.96E-06 | 80.89 (78.65–83.14) | AC092570.4 | intergenic |
| 19 | rs8107081 | T | C | 0.16 | 0.24 | 0.00 | 4.27E-06 | NA | NLRP7 | intronic |
| 2 | rs10192833 | G | A | 0.16 | 0.24 | 0.00 | 5.02E-06 | NA | SLC8A1 | intronic |
| 8 | rs13251621 | G | T | 0.86 | 0.95 | 0.65 | 5.57E-06 | 14.17 (12.86–15.47) | CHD7 | intergenic |
| 5 | rs73766662 | T | C | 0.91 | 0.98 | 0.75 | 5.78E-06 | NA | RP11-434D9.1 | ncRNA_intronic |
| 12 | rs75642727 | C | T | 0.50 | 0.62 | 0.23 | 6.59E-06 | 7.73 (6.66–8.8) | ITPR2 | intronic |
| 14 | rs75452476 | C | T | 0.66 | 0.77 | 0.42 | 6.65E-06 | 9.7 (8.48–10.92) | RPL15P2 | intergenic |
| 16 | rs72812239 | C | T | 0.31 | 0.42 | 0.07 | 7.31E-06 | 11.67 (10.31–13.03) | RP4-536B24.3 | ncRNA_intronic |
| 20 | rs6080877 | T | C | 0.13 | 0.18 | 0.00 | 8.15E-06 | NA | AL035045.1 | intergenic |
| 11 | rs35268671 | C | G | 0.79 | 0.89 | 0.58 | 8.77E-06 | 10.83 (9.63–12.03) | RP11-266A24.1 | intergenic |
| 18 | rs1010003 | A | G | 0.14 | 0.20 | 0.01 | 8.87E-06 | NA | RP11-689C9.1 | ncRNA_intronic |
| 13 | rs9519332 | A | G | 0.40 | 0.52 | 0.14 | 9.01E-06 | 8.25 (7.11–9.39) | ATP6V1G1P7 | intergenic |

**Table S5. Table of genomic risk loci for gabapentin side-effect burden at a nominal association threshold ($p < 1 \times 10^{-5}$). Related to Figure S5.**

Note: OR (95% CI) is not presented where EAF in cases or controls is <0.05 or >0.95. EA: effect allele; NEA: non-effect allele; EAF: effect allele frequency; OR: odds ratio; CI: confidence interval.

| | |
|---|---|
| Analysis Type: | PANTHER Overrepresentation Test (Released 20221013) |
| Annotation Version and Release Date: | PANTHER version 17·0 Released 2022-02-22 |
| Analyzed List: | Client Text Box Input (Homo sapiens) |
| Reference List: | Homo sapiens (all genes in database) |
| Test Type: | FISHER |
| Correction: | FDR |

| PANTHER Pathways | Homo sapiens - REFLIST (20589) | Client Text Box Input (54) | Client Text Box Input (expected) | Client Text Box Input (over/under) | Client Text Box Input (fold Enrichment) | Client Text Box Input (raw P-value) | Client Text Box Input (FDR) |
|---|---|---|---|---|---|---|---|
| Cadherin signaling pathway (P00012) | 166 | 17 | 0·44 | + | 39·05 | 2·14E-22 | 3·43E-20 |
| Wnt signaling pathway (P00057) | 314 | 18 | 0·82 | + | 21·86 | 1·88E-19 | 1·50E-17 |
| Unclassified (UNCLASSIFIED) | 17971 | 32 | 47·13 | - | 0·68 | 2·62E-07 | 1·39E-05 |

**Table S6. PANTHER Pathway statistical overrepresentation analysis of genes mapped to gabapentin pain response. Related to Figure S5.**

Results of PANTHER Pathway statistical overrepresentation analysis of genes mapped to gabapentin pain response at a nominal association threshold ($p < 1 \times 10^{-5}$). 54 genes mapped and 25 unrecognised for gabapentin pain response.
